# Supplementary material for: LNS8801: An Enantiomerically Pure Agonist of the G Protein–Coupled Estrogen Receptor Suitable for Clinical Development
Source: Cancer Res Commun. 2025 Apr 4;5(4):556–68. doi: 10.1158/2767-9764.CRC-24-0632 (PMC11969138; doi:10.1158/2767-9764.CRC-24-0632)
Supplement: Supplementary Data — Supplemental Figure Legends [file crc-24-0632_supplementary_data_suppsfl.docx]

**Supplemental Figure Legends**

*Supplemental Figure 1: Validation of LNS8801 activity and necessity of GPER in HL-60 cells. (A) Dose response of cAMP assay in HL-60 cells treated with G-1, LNS8801, and LNS8812 relative to control, n=3. * denotes*  p<0.005 and ** denotes p<0.0005 *by one-way ANOVA with multiple comparisions (Tukey)*. *(B) Western blot demonstrating GPER depletion by anti-GPER siRNA in HL-60 cells. (C) cAMP assay of HL-60 cells with and without GPER siRNA depletion treated with G-1, LNS8801, and LNS8812, n=3. * denotes*  p<0.05 and ** denotes p<0.005 *by one-way ANOVA with multiple comparisions (Tukey)*.

*Supplemental Figure 2: LNS8801 is active in the WM46 human xenograft model. (A) Tumor volumes and (B) Kaplan-Meier survival curves measured over time in 2838c3 PDAC-bearing mice treated with orally delivered vehicle and LNS8801 at 1 mg/kg, significance by log-rank (Mantel-Cox), n=5 per group.*

*Supplemental Figure 3: CRISPR-Cas9 depletion of GPER in human melanocytes. (A) Agarose gel of PCR-amplified GPER from genomic DNA in control or GPER-depleted cells via CRISPR-Cas9. (B) Sanger sequencing of cells targeted with control CRISPR-Cas9. (C) Sanger sequencing of cells targeted with GPER CRISPR-Cas9.*

*Supplemental Table 1: Off-target binding of LNS8801 and LNS8812 using Eurofins Discover X.*

*Supplemental Table 2: Log-rank (Mantel-Cox) all comparisions of data in Figure 2 and 4*
