# Supplementary figures and images for: LNS8801: An Enantiomerically Pure Agonist of the G Protein–Coupled Estrogen Receptor Suitable for Clinical Development
Source: Cancer Res Commun. 2025 Apr 4;5(4):556–68. doi: 10.1158/2767-9764.CRC-24-0632 (PMC11969138; doi:10.1158/2767-9764.CRC-24-0632)

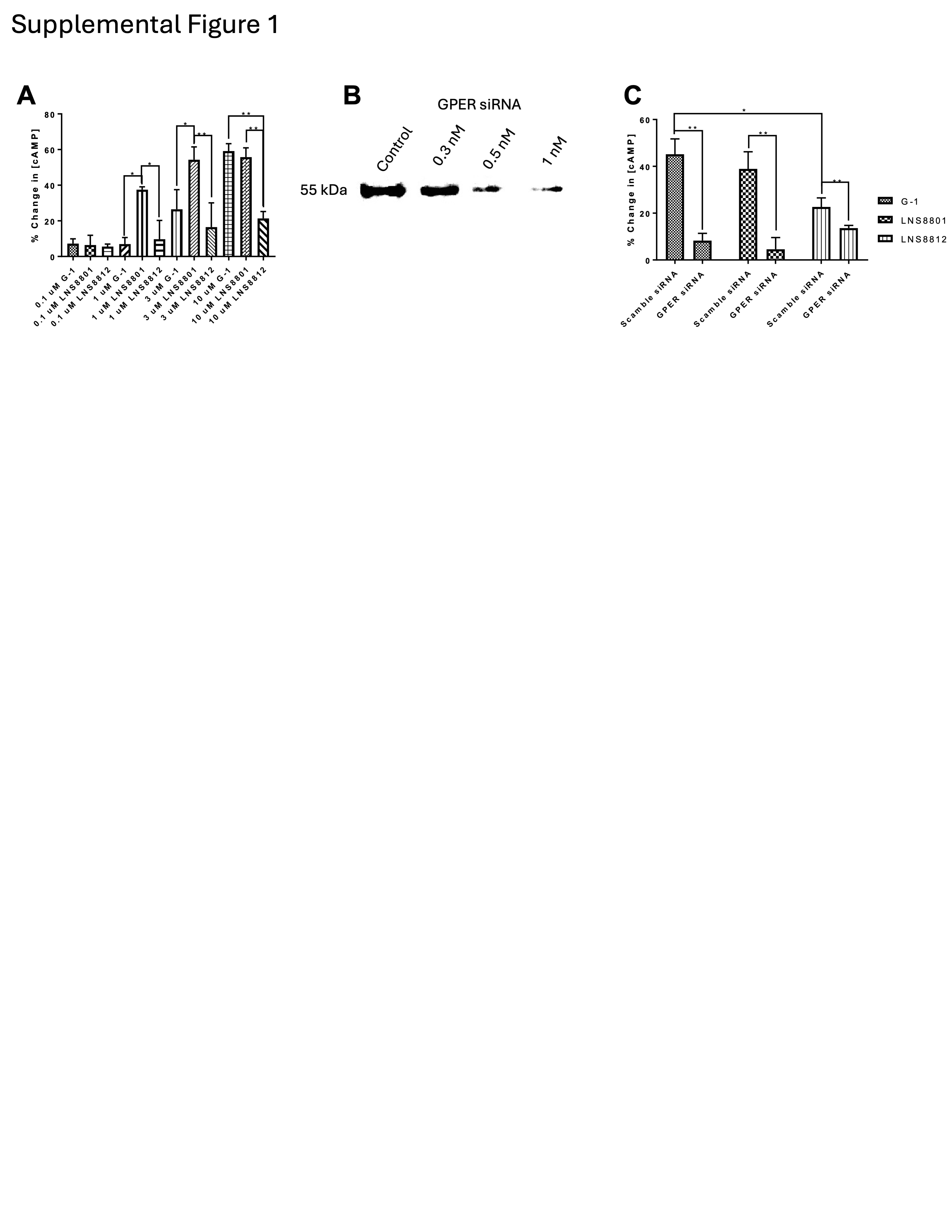

Supplement: Supplemental Figure 1 — S1. Validation of LNS8801 activity and necessity of GPER in HL-60 cells. [file crc-24-0632_supplemental_figure_1_suppsf1.png]

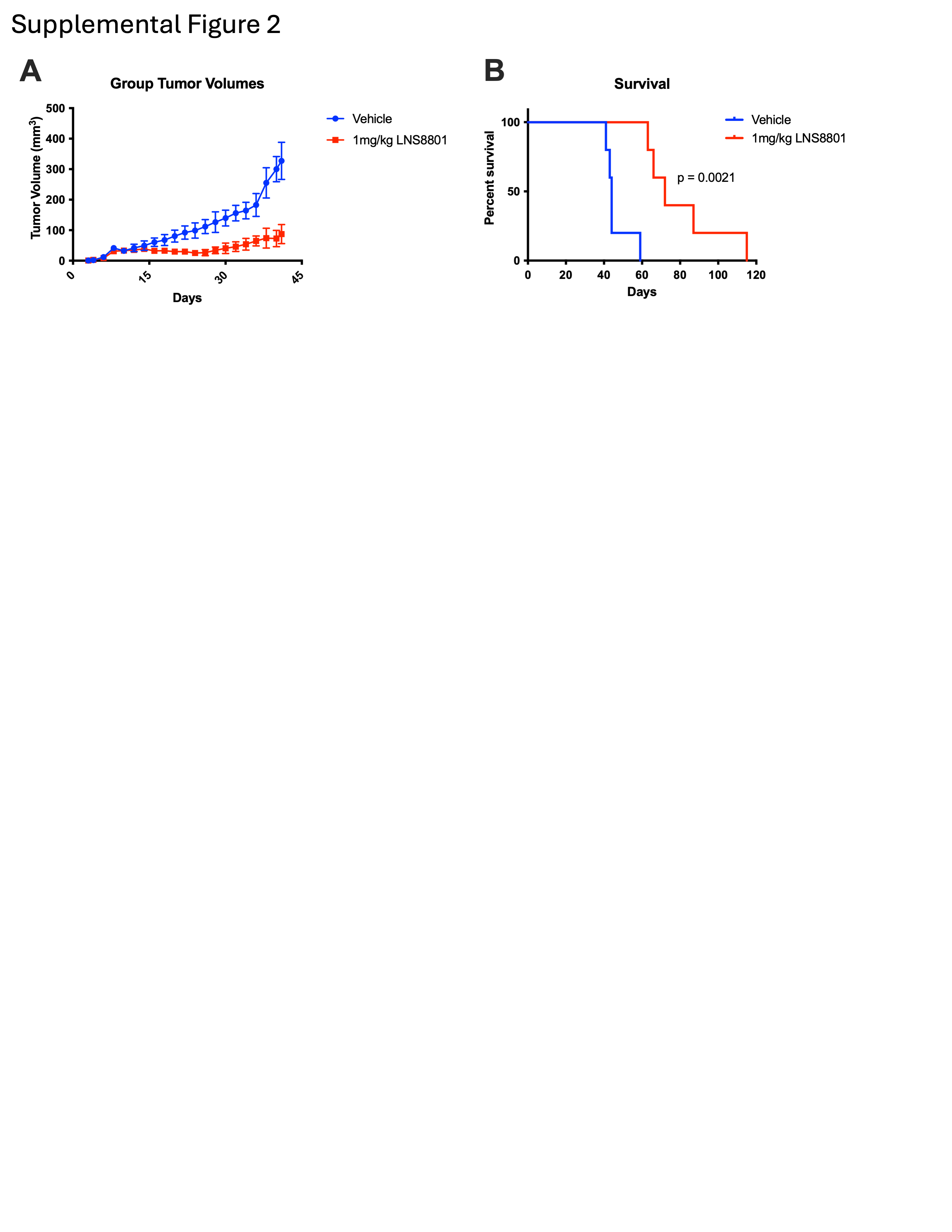

Supplement: Supplemental Figure 2 — S2. LNS8801 is active in the WM46 human xenograft model. [file crc-24-0632_supplemental_figure_2_suppsf2.png]

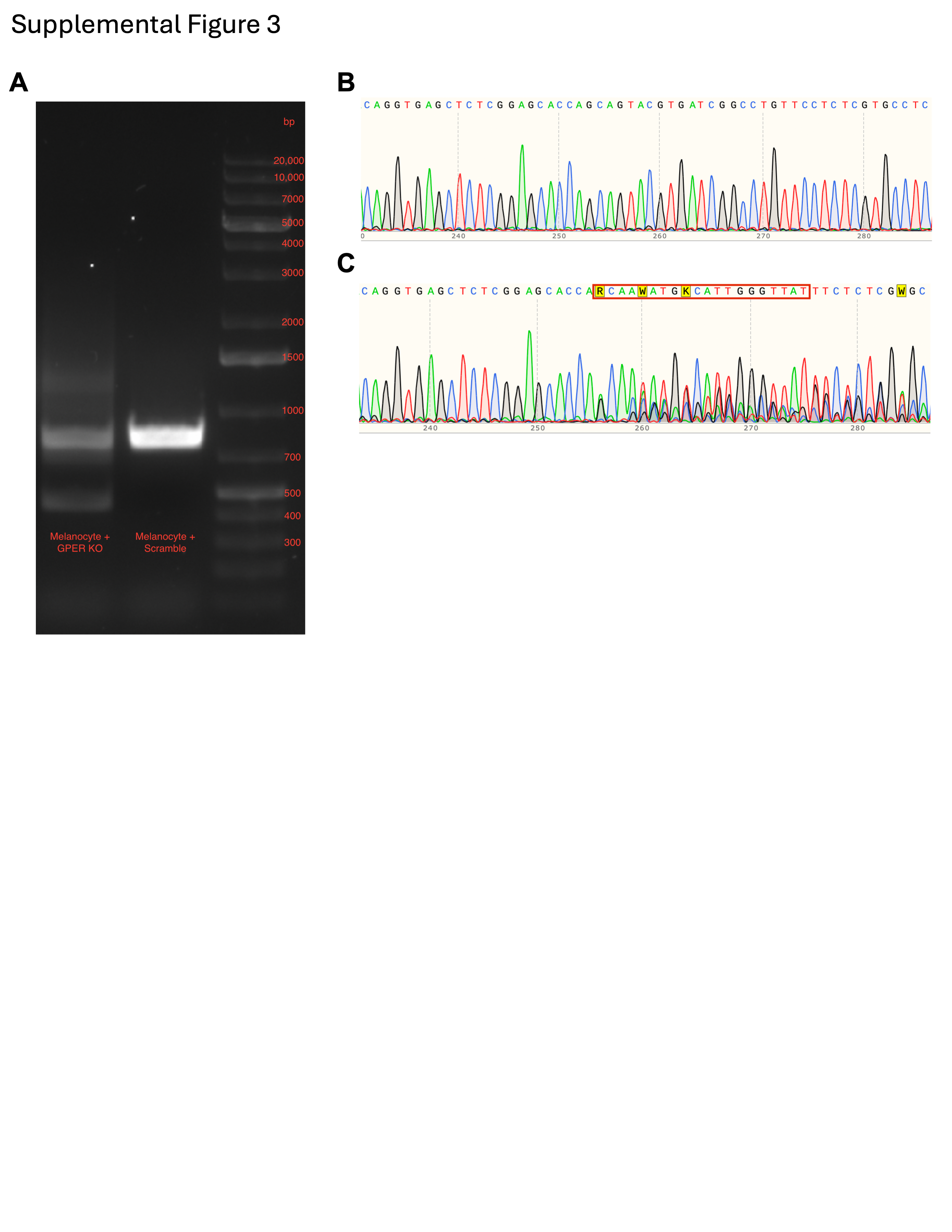

Supplement: Supplemental Figure 3 — S3. CRISPR-Cas9 depletion of GPER in human melanocytes. [file crc-24-0632_supplemental_figure_3_suppsf3.png]

Supplemental Table 1


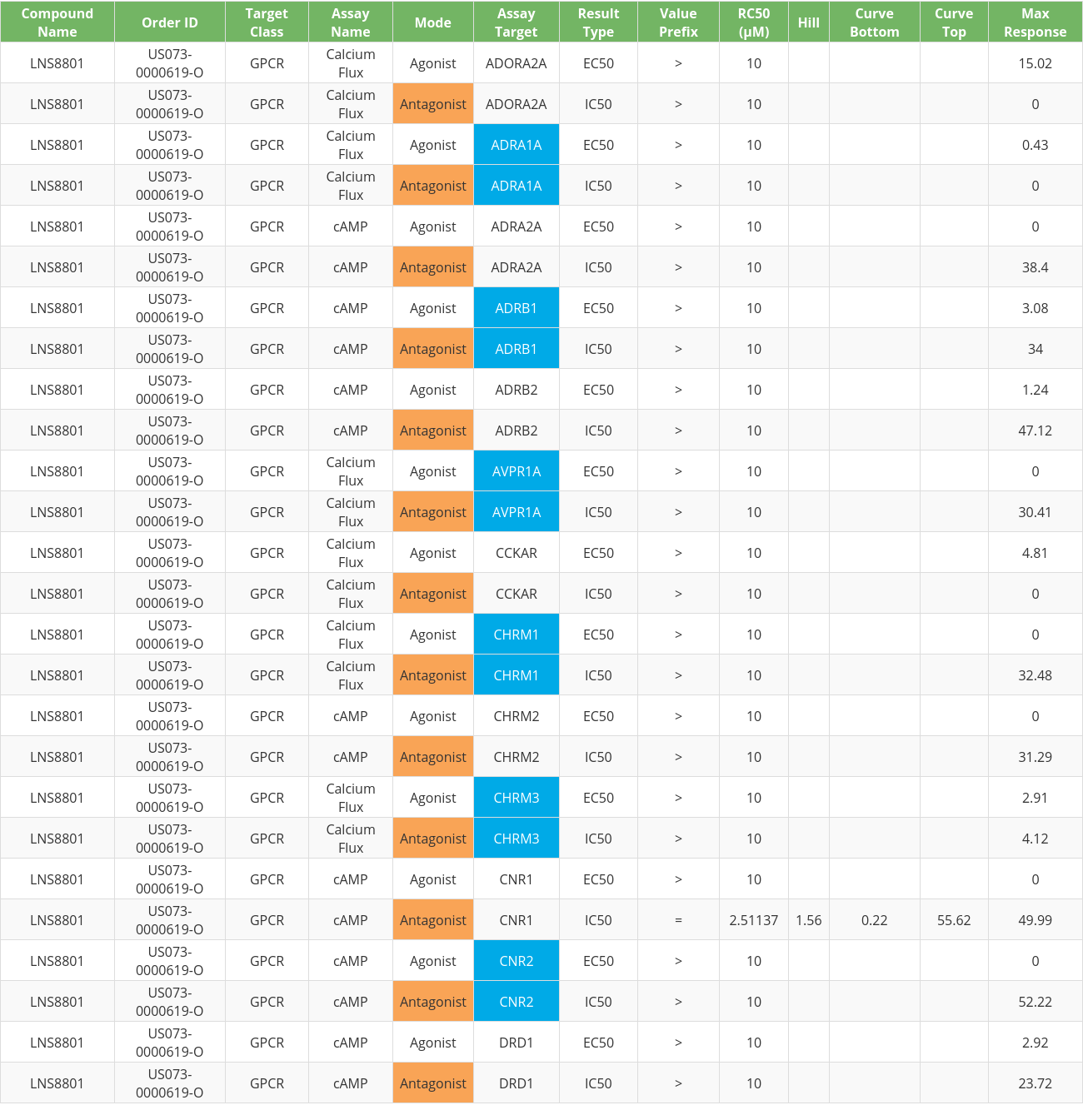


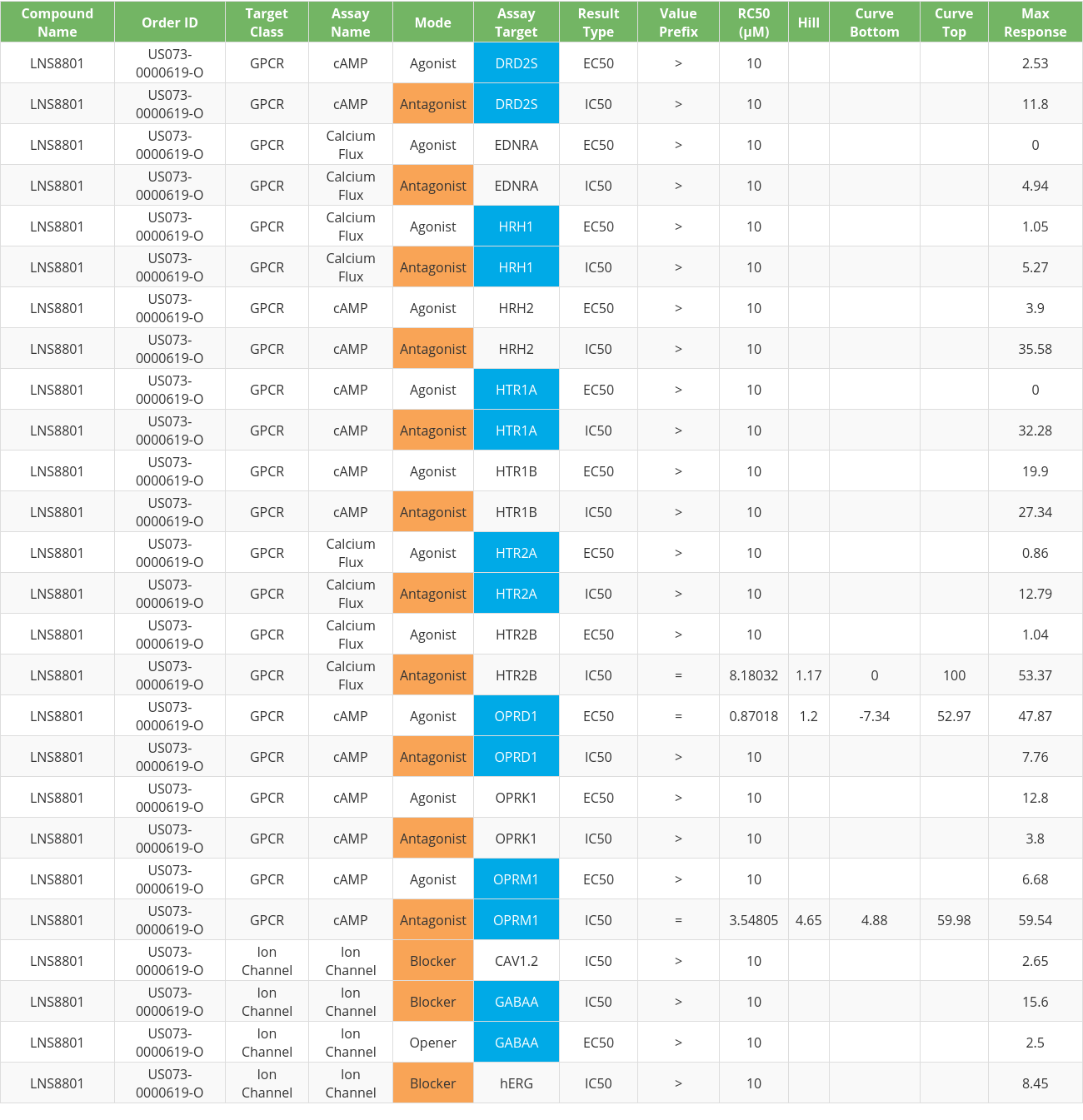


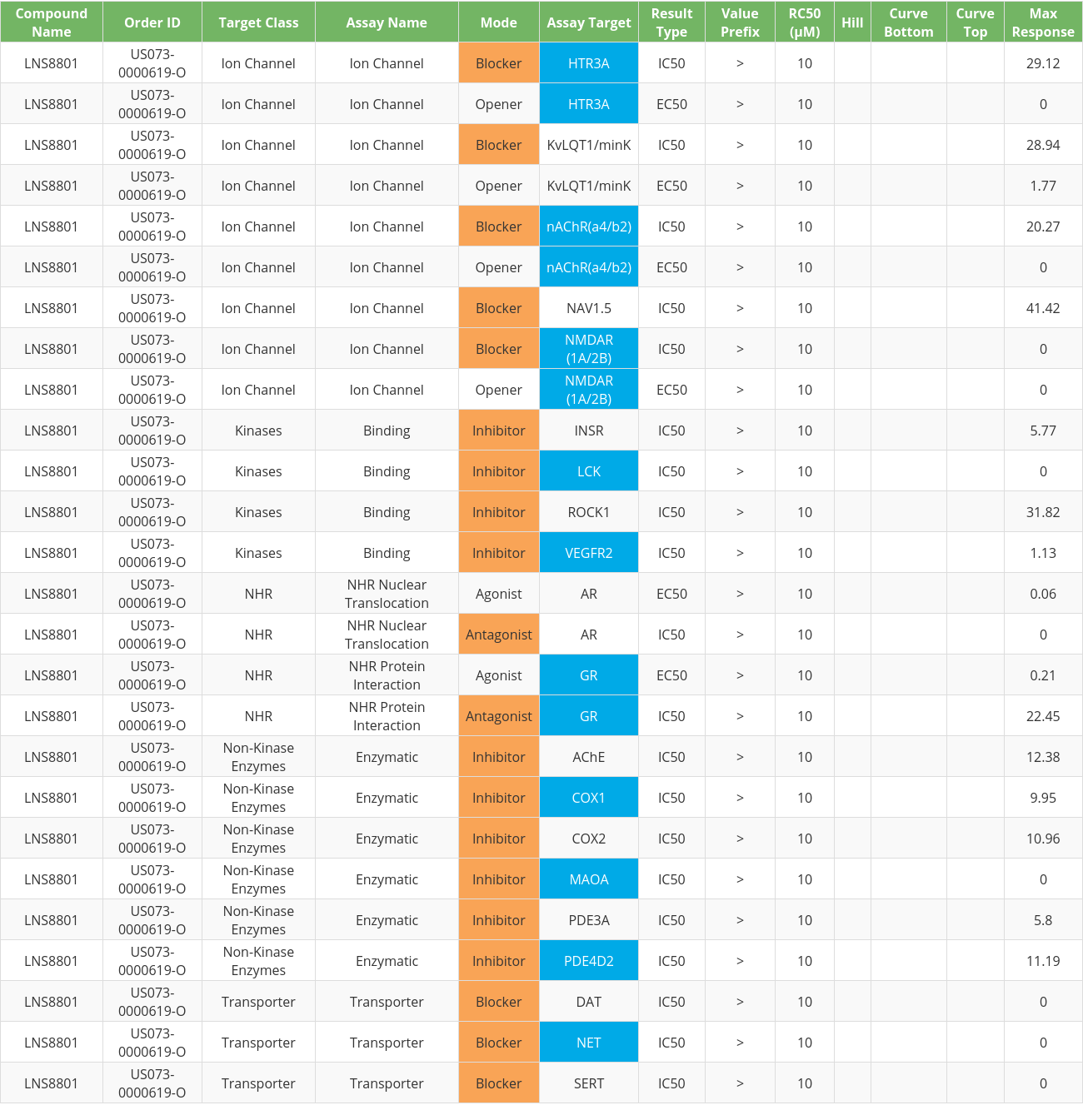


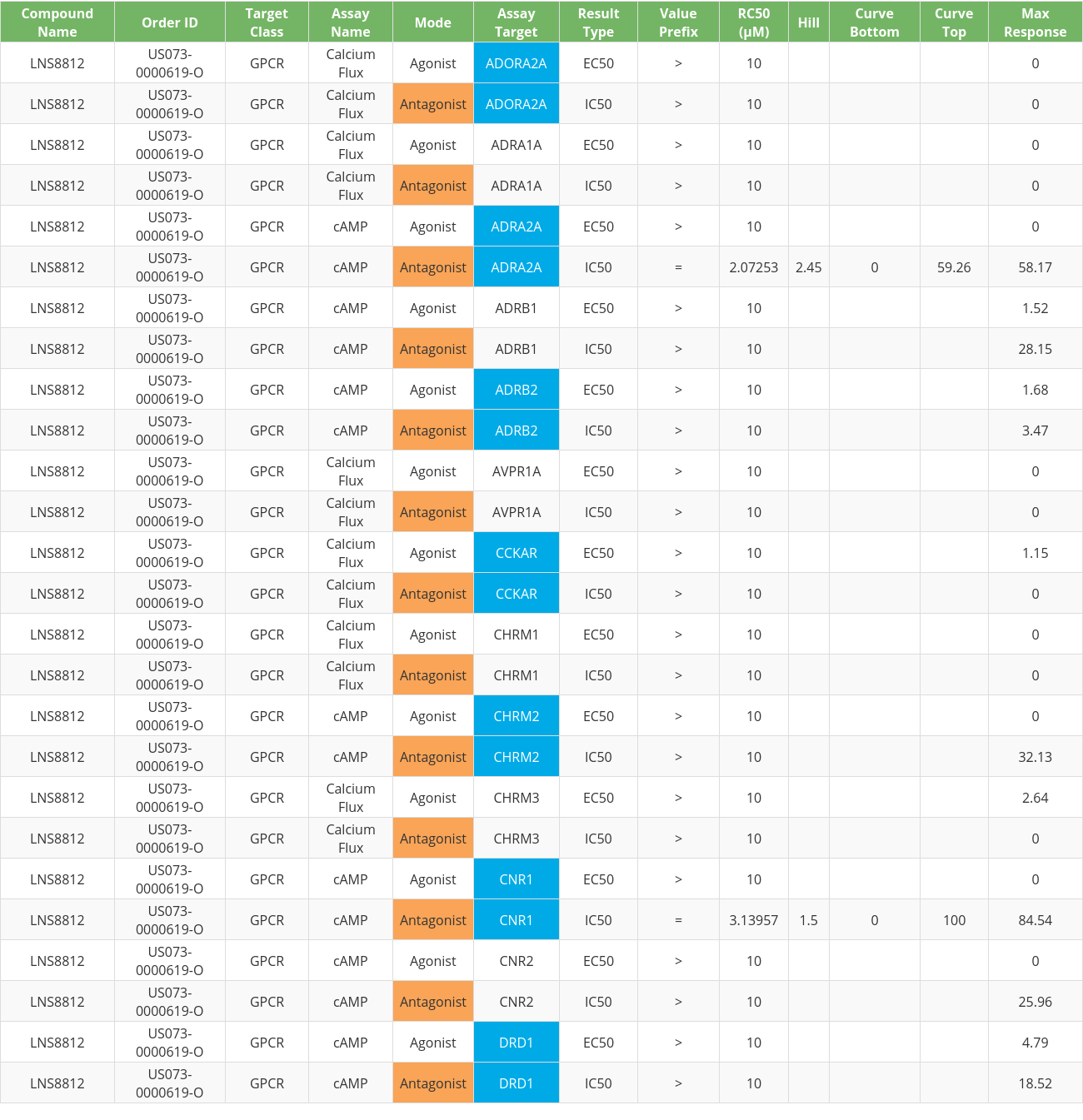


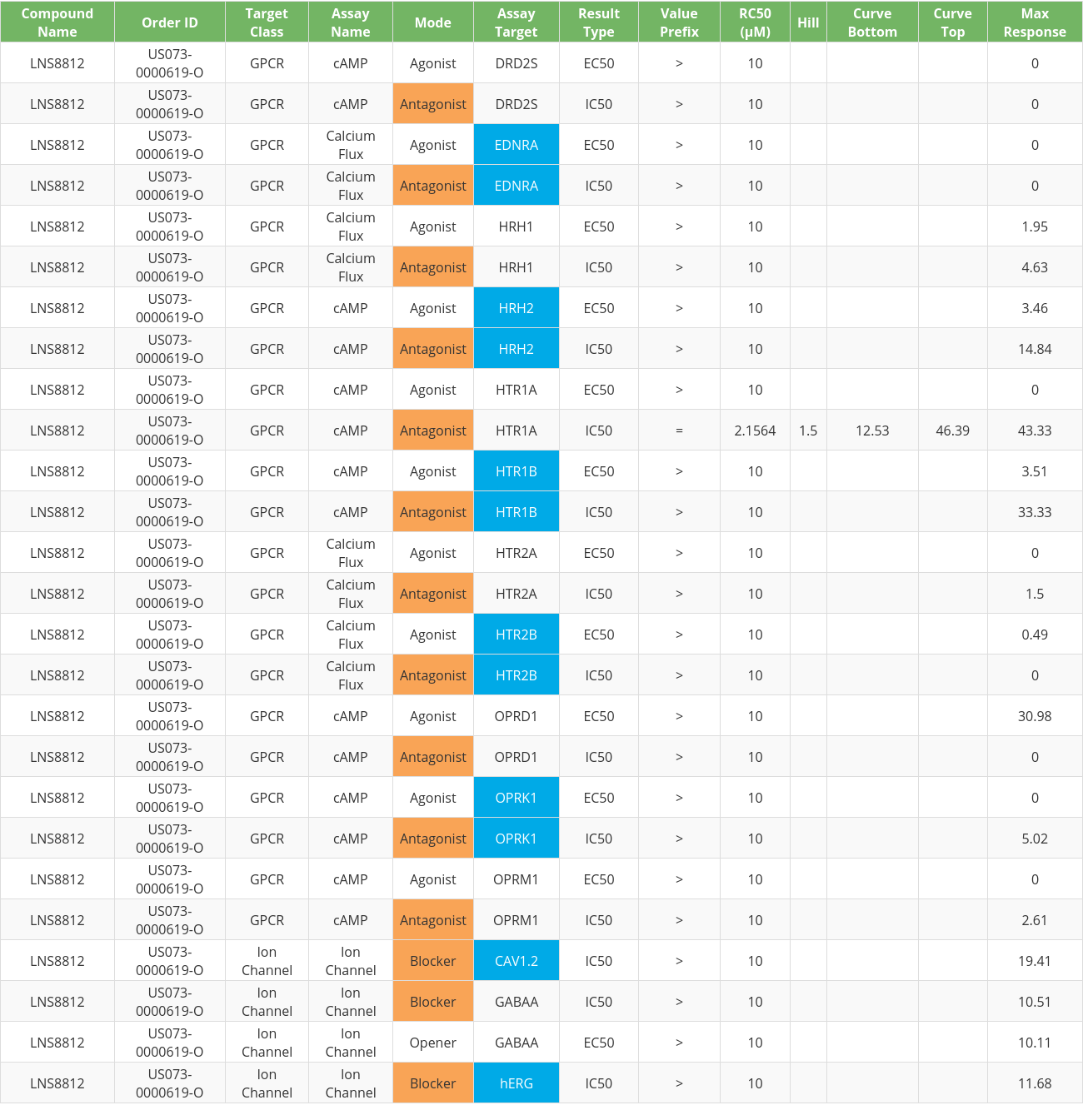


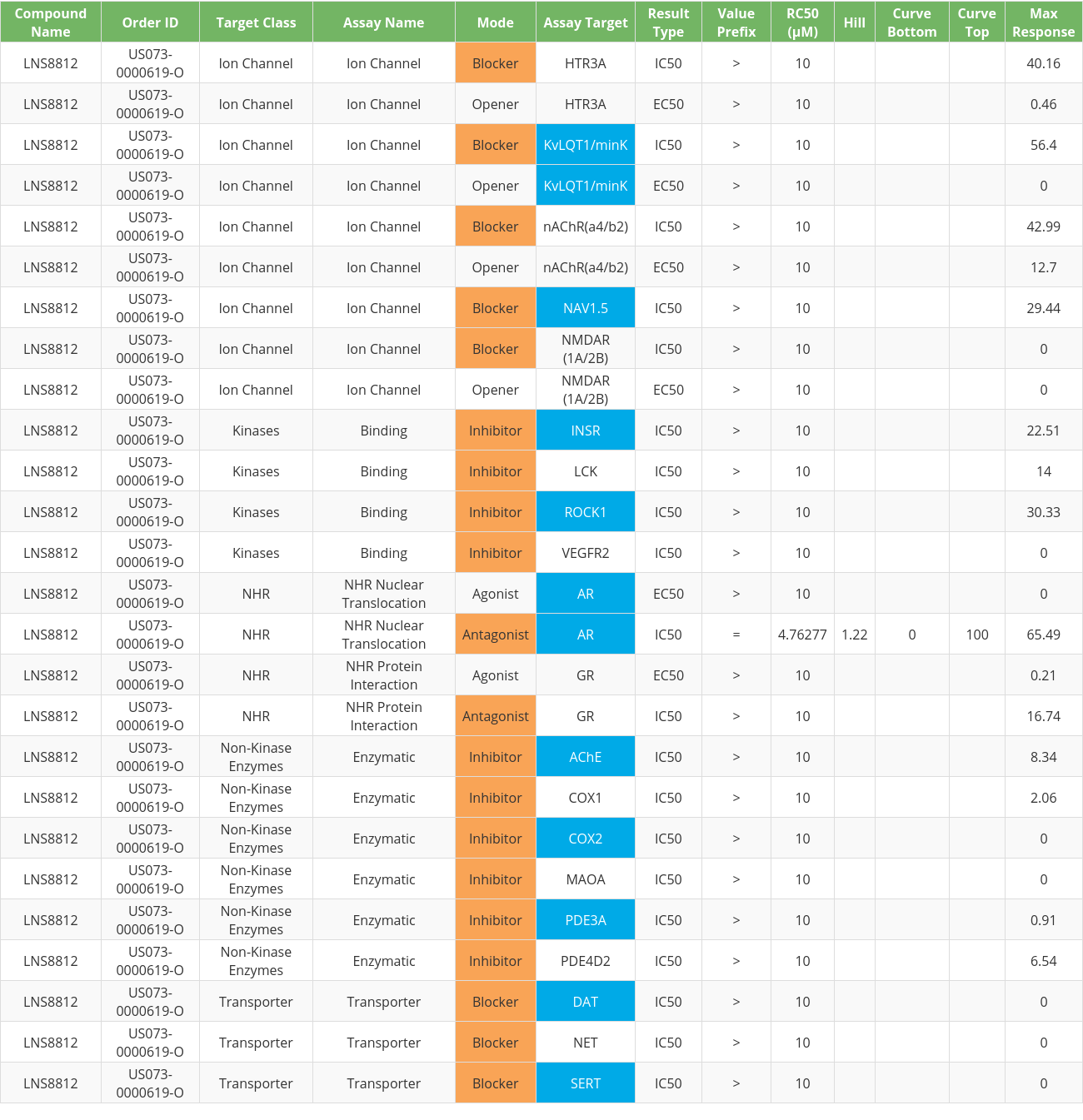

Supplement: Supplemental Table 1 — Off-target binding of LNS8801 and LNS8812 using Eurofins Discover X. [file crc-24-0632_supplemental_table_1_suppst1.docx]
