# Supplementary material for: LNS8801: An Enantiomerically Pure Agonist of the G Protein–Coupled Estrogen Receptor Suitable for Clinical Development
Source: Cancer Res Commun. 2025 Apr 4;5(4):556–68. doi: 10.1158/2767-9764.CRC-24-0632 (PMC11969138; doi:10.1158/2767-9764.CRC-24-0632)
Supplement: Supplemental Table 2 — Log-rank (Mantel-Cox) all comparisions of data in Figure 2 and 4. [file crc-24-0632_supplemental_table_2_suppst2.docx]

Supplemental Table 2

| **Figure 2B** | Vehicle | 1mg/kg G-1 | 1mg/kg LNS8801 | 1mg/kg LNS8812 |
| --- | --- | --- | --- | --- |
| Vehicle | 1 | **0.0181** | **0.0084** | 0.4482 |
| 1mg/kg G-1 |  | 1 | **0.0396** | **0.0086** |
| 1mg/kg LNS8801 |  |  | 1 | **0.0048** |
| 1mg/kg LNS8812 |  |  |  | 1 |
|  |  |  |  |  |
| **Figure 2D** | Control | Subcutaneous | Oral soluble | Oral insoluble |
| Control | 1 | **0.0023** | **0.0052** | **0.0023** |
| Subcutaneous |  | 1 | 0.917 | 0.3903 |
| Oral soluble |  |  | 1 | **0.04** |
| Oral insoluble |  |  |  | 1 |
|  |  |  |  |  |
| **Figure 2F** | Vehicle | 0.01mg/kg | 0.1mg/kg | 1mg/kg |
| Vehicle | 1 | 0.773 | **0.0019** | **0.0019** |
| 0.01mg/kg |  | 1 | **0.0029** | **0.0029** |
| 0.1mg/kg |  |  | 1 | 0.6198 |
| 1mg/kg |  |  |  | 1 |
|  |  |  |  |  |
| **Figure 4D** | GPER WT Veh | GPER WT LNS | GPER KO Veh | GPER KO LNS |
| GPER WT Veh | 1 | **0.0027** | 0.1967 | 0.1967 |
| GPER WT LNS |  | 1 | **0.0068** | **0.0082** |
| GPER KO Veh |  |  | 1 | 0.3173 |
| GPER KO LNS |  |  |  | 1 |
